# Supplementary material for: Empirical methods for controlling false positives and estimating confidence in ChIP-Seq peaks
Source: BMC Bioinformatics. 2008 Dec 5;9:523. doi: 10.1186/1471-2105-9-523 (PMC2628906; doi:10.1186/1471-2105-9-523)
Supplement: Additional File 1 — A variety of html documents from the USeq web site detailing the available applications, their best usage, output file type descriptions, command line menus, etc. [file 1471-2105-9-523-S1.zip › USeqUserGuides/JavaDoc.zip!/JavaDoc/bioroot/antibody/AntibodyBase.html]

AntibodyBase


|  |  |  |
| --- | --- | --- |
| **Overview** \| **Package** \| **Class** \| **Use** \| **Tree** \| **Deprecated** \| **Index** \| **Help** | |  |
| PREV CLASS   **NEXT CLASS** | **FRAMES**    **NO FRAMES**     **All Classes** |  |
| SUMMARY: NESTED \| FIELD \| CONSTR \| METHOD | DETAIL: FIELD \| CONSTR \| METHOD |  |


---


## bioroot.antibody Class AntibodyBase

```
java.lang.Object
  javax.servlet.GenericServlet
      javax.servlet.http.HttpServlet
          bioroot.antibody.AntibodyBase
```

**All Implemented Interfaces:**: java.io.Serializable, javax.servlet.Servlet, javax.servlet.ServletConfig

---

``` public class AntibodyBase extends javax.servlet.http.HttpServlet ```

**Author:**
:   nix
    Contains static methods for Plasmids

**See Also:**: Serialized Form

---

| **Field Summary** | |
| --- | --- |
| `static java.lang.String` | `antibodyPrepFeatureNames`             Used to see if any antibody preps are called for spreadsheet. |
| `static java.lang.String[]` | `searchHeadings`             Use to make a hash of column heading in which to search, name then the actual search column name. |
| `static java.lang.String[]` | `stringFeatures`             Use to make a default antibody Feature[]. |
| `static java.lang.String` | `useDilutionFeatureNames`             Used to see if any use dilutions are called for spreadsheet. |


**Constructor Summary**

`AntibodyBase()`


| **Method Summary** | |
| --- | --- |
| `static void` | `addPlasmid(AntibodyBean bean, java.util.HashMap nameValue, DBUtil bioRoot, UserBean userBean)` |
| `static void` | `addStrain(AntibodyBean bean, java.util.HashMap nameValue, DBUtil bioRoot, UserBean userBean)` |
| `static int` | `deleteAntibodyPrepIndex(AntibodyBean a, DBUtil bioRoot, java.util.HashMap m)`             Scans a hashmap for an antibody prep to delete returns the index number |
| `static java.lang.String` | `deleteUseDilutionIndex(AntibodyBean a, DBUtil bioRoot, java.util.HashMap m)`             Scans a hashmap for a use dilution to delete returns the index string or null. |
| `void` | `doGet(javax.servlet.http.HttpServletRequest request, javax.servlet.http.HttpServletResponse response)` |
| `void` | `doPost(javax.servlet.http.HttpServletRequest request, javax.servlet.http.HttpServletResponse response)` |
| `static java.lang.String` | `getAntibodyForm(AntibodyBean ab, DBUtil bioRoot, UserBean userBean)`             Returns an antibody form filled with the values in the bean. |
| `static void` | `getAntibodyPrepForm(AntibodyPrep a, DBUtil bioRoot, java.lang.StringBuffer b, int index)` |
| `static void` | `getAntibodyPrepForms(AntibodyBean ab, DBUtil bioRoot, java.lang.StringBuffer b)` |
| `static void` | `getAntibodyPrepReport(AntibodyPrep a, DBUtil bioRoot, java.lang.StringBuffer b, int index, boolean commonForm)` |
| `static void` | `getAntibodyPrepReports(AntibodyBean ab, DBUtil bioRoot, java.lang.StringBuffer b, boolean commonForm)` |
| `static java.lang.String` | `getAntibodyReport(AntibodyBean ab, DBUtil bioRoot, UserBean user, boolean commonForm)`             Returns an antibody report filled with the values in the bean. |
| `static java.lang.String[]` | `getSearchColumnNames()`             Returns a list of the search column names used in an SQL statment , ie Oligo.name, 1/2 the searchHash. |
| `static java.util.LinkedHashMap` | `getSearchHash()` |
| `static java.lang.String[]` | `getSearchNames()` |
| `static void` | `getUseDilutionForm(UseDilution u, java.lang.StringBuffer b, int index, int apbIndex, boolean lastForm)`             Returns a UseDilutionNotes where the names are apended with apbIndex.index ie 0.0, 0.1, 0.2,,1.0,1.1... |
| `static void` | `getUseDilutionForms(AntibodyPrep a, DBUtil bioRoot, java.lang.StringBuffer b, int apbIndex)`             Pumps out useDilution forms, should always have one with each Prep |
| `static void` | `getUseDilutionReport(UseDilution u, java.lang.StringBuffer b, int index, int apbIndex)` |
| `static void` | `getUseDilutionReports(AntibodyPrep a, DBUtil bioRoot, java.lang.StringBuffer b, int apbIndex, boolean commonForm)`             Pumps out useDilution reports |
| `static int` | `newUseDilutionIndex(AntibodyBean a, DBUtil bioRoot, java.util.HashMap m)`             Scans a hashmap for a newUseDilution selection, returns the index number |
| `static void` | `removePlasmid(AntibodyBean bean, java.util.HashMap nameValue, DBUtil bioRoot)` |
| `static void` | `removeStrain(AntibodyBean bean, java.util.HashMap nameValue, DBUtil bioRoot)` |
| `static java.lang.String` | `renameFile(java.lang.String oldFileName, java.lang.String directory)`             Copies an old file and renames it to tempRndmWrd\_name. |
| `static AntibodyBean[]` | `reverseAntibodyBeans(AntibodyBean[] beans)`             Reverses the order of an array of PlasmidBeans. |
| `static Feature[][]` | `splitAntibodyFeatures(Feature[] all)`             Returns arrays of Features where the F[0] = antibody, F[1]=abPrep, F[2]=useDil specific features. |
| `static java.lang.String` | `writeTempFile(java.lang.Object fileItemPoss, javax.servlet.http.HttpServletRequest request, java.lang.String directory)`             Looks for an uploaded file, writes it to the directory with a tempRNDMWord\_FileName, will return the name, or null, or 'Too big' |

**Methods inherited from class javax.servlet.http.HttpServlet**

`service`

**Methods inherited from class javax.servlet.GenericServlet**

`destroy, getInitParameter, getInitParameterNames, getServletConfig, getServletContext, getServletInfo, getServletName, init, init, log, log`

**Methods inherited from class java.lang.Object**

`equals, getClass, hashCode, notify, notifyAll, toString, wait, wait, wait`

**Field Detail**

### stringFeatures

```
public static java.lang.String[] stringFeatures
```

:   Use to make a default antibody Feature[]. Watch order, stops when it finds a false.
    Be sure to reflect any changes here to the featureName Strings below.

---


### antibodyPrepFeatureNames

```
public static final java.lang.String antibodyPrepFeatureNames
```

:   Used to see if any antibody preps are called for spreadsheet.
    Should match all of the feature names for antibodyPreps.
    Keep the last comma.

    **See Also:**: Constant Field Values

---


### useDilutionFeatureNames

```
public static final java.lang.String useDilutionFeatureNames
```

:   Used to see if any use dilutions are called for spreadsheet.
    Should match all of the feature names for use dilutions.
    Keep the last comma.

    **See Also:**: Constant Field Values

---


### searchHeadings

```
public static final java.lang.String[] searchHeadings
```

:   Use to make a hash of column heading in which to search, name then the actual search column name.


**Constructor Detail**

### AntibodyBase

```
public AntibodyBase()
```


**Method Detail**

### splitAntibodyFeatures

```
public static Feature[][] splitAntibodyFeatures(Feature[] all)
```

:   Returns arrays of Features where the F[0] = antibody, F[1]=abPrep, F[2]=useDil
    specific features. For displaying in the Antibody View Preferences

---


### getSearchHash

```
public static java.util.LinkedHashMap getSearchHash()
```

---


### getSearchNames

```
public static java.lang.String[] getSearchNames()
```

---


### getSearchColumnNames

```
public static java.lang.String[] getSearchColumnNames()
```

:   Returns a list of the search column names used in an SQL statment , ie Oligo.name, 1/2 the searchHash.

---


### doGet

```
public void doGet(javax.servlet.http.HttpServletRequest request,
                  javax.servlet.http.HttpServletResponse response)
           throws javax.servlet.ServletException,
                  java.io.IOException
```

:   **Overrides:**: `doGet` in class `javax.servlet.http.HttpServlet`
:   **Throws:**: `javax.servlet.ServletException`: `java.io.IOException`

---


### doPost

```
public void doPost(javax.servlet.http.HttpServletRequest request,
                   javax.servlet.http.HttpServletResponse response)
            throws javax.servlet.ServletException,
                   java.io.IOException
```

:   **Overrides:**: `doPost` in class `javax.servlet.http.HttpServlet`
:   **Throws:**: `javax.servlet.ServletException`: `java.io.IOException`

---


### addStrain

```
public static void addStrain(AntibodyBean bean,
                             java.util.HashMap nameValue,
                             DBUtil bioRoot,
                             UserBean userBean)
```

---


### removeStrain

```
public static void removeStrain(AntibodyBean bean,
                                java.util.HashMap nameValue,
                                DBUtil bioRoot)
```

---


### addPlasmid

```
public static void addPlasmid(AntibodyBean bean,
                              java.util.HashMap nameValue,
                              DBUtil bioRoot,
                              UserBean userBean)
```

---


### removePlasmid

```
public static void removePlasmid(AntibodyBean bean,
                                 java.util.HashMap nameValue,
                                 DBUtil bioRoot)
```

---


### deleteUseDilutionIndex

```
public static java.lang.String deleteUseDilutionIndex(AntibodyBean a,
                                                      DBUtil bioRoot,
                                                      java.util.HashMap m)
```

:   Scans a hashmap for a use dilution to delete returns the index string or null.

---


### deleteAntibodyPrepIndex

```
public static int deleteAntibodyPrepIndex(AntibodyBean a,
                                          DBUtil bioRoot,
                                          java.util.HashMap m)
```

:   Scans a hashmap for an antibody prep to delete returns the index number

---


### newUseDilutionIndex

```
public static int newUseDilutionIndex(AntibodyBean a,
                                      DBUtil bioRoot,
                                      java.util.HashMap m)
```

:   Scans a hashmap for a newUseDilution selection, returns the index number

---


### reverseAntibodyBeans

```
public static AntibodyBean[] reverseAntibodyBeans(AntibodyBean[] beans)
```

:   Reverses the order of an array of PlasmidBeans.

---


### getAntibodyForm

```
public static java.lang.String getAntibodyForm(AntibodyBean ab,
                                               DBUtil bioRoot,
                                               UserBean userBean)
```

:   Returns an antibody form filled with the values in the bean. Must add a form entry. ie
    + "

    " and start and stop HTML stuff

---


### getAntibodyPrepForms

```
public static void getAntibodyPrepForms(AntibodyBean ab,
                                        DBUtil bioRoot,
                                        java.lang.StringBuffer b)
```

---


### getAntibodyPrepForm

```
public static void getAntibodyPrepForm(AntibodyPrep a,
                                       DBUtil bioRoot,
                                       java.lang.StringBuffer b,
                                       int index)
```

---


### getUseDilutionForms

```
public static void getUseDilutionForms(AntibodyPrep a,
                                       DBUtil bioRoot,
                                       java.lang.StringBuffer b,
                                       int apbIndex)
```

:   Pumps out useDilution forms, should always have one with each Prep

---


### getUseDilutionForm

```
public static void getUseDilutionForm(UseDilution u,
                                      java.lang.StringBuffer b,
                                      int index,
                                      int apbIndex,
                                      boolean lastForm)
```

:   Returns a|  |  |
    | --- | --- |
    |Use Dilution Notes where the names are apended with apbIndex.index ie 0.0, 0.1, 0.2,,1.0,1.1... | |

---


### getAntibodyReport

```
public static java.lang.String getAntibodyReport(AntibodyBean ab,
                                                 DBUtil bioRoot,
                                                 UserBean user,
                                                 boolean commonForm)
```

:   Returns an antibody report filled with the values in the bean.

---


### getAntibodyPrepReports

```
public static void getAntibodyPrepReports(AntibodyBean ab,
                                          DBUtil bioRoot,
                                          java.lang.StringBuffer b,
                                          boolean commonForm)
```

---


### getAntibodyPrepReport

```
public static void getAntibodyPrepReport(AntibodyPrep a,
                                         DBUtil bioRoot,
                                         java.lang.StringBuffer b,
                                         int index,
                                         boolean commonForm)
```

---


### getUseDilutionReports

```
public static void getUseDilutionReports(AntibodyPrep a,
                                         DBUtil bioRoot,
                                         java.lang.StringBuffer b,
                                         int apbIndex,
                                         boolean commonForm)
```

:   Pumps out useDilution reports

---


### getUseDilutionReport

```
public static void getUseDilutionReport(UseDilution u,
                                        java.lang.StringBuffer b,
                                        int index,
                                        int apbIndex)
```

---


### writeTempFile

```
public static java.lang.String writeTempFile(java.lang.Object fileItemPoss,
                                             javax.servlet.http.HttpServletRequest request,
                                             java.lang.String directory)
```

:   Looks for an uploaded file, writes it to the directory with a tempRNDMWord\_FileName,
    will return the name, or null, or 'Too big'

---


### renameFile

```
public static java.lang.String renameFile(java.lang.String oldFileName,
                                          java.lang.String directory)
```

:   Copies an old file and renames it to tempRndmWrd\_name.
    Returns null problems.


---


|  |  |  |
| --- | --- | --- |
| **Overview** \| **Package** \| **Class** \| **Use** \| **Tree** \| **Deprecated** \| **Index** \| **Help** | |  |
| PREV CLASS   **NEXT CLASS** | **FRAMES**    **NO FRAMES**     **All Classes** |  |
| SUMMARY: NESTED \| FIELD \| CONSTR \| METHOD | DETAIL: FIELD \| CONSTR \| METHOD |  |


---
